# Mode de révélation particulier d'une tumeur brune chez une patiente hémodialysée chronique

**DOI:** 10.11604/pamj.2014.18.223.4051

**Published:** 2014-07-17

**Authors:** Ryme El Harraqui, Ilham Karimi, Abdeljalil Chemlal, Fatiha Alaoui Ismaili, Intissar Haddiya

**Affiliations:** 1 Service de Néphrologie-Dialyse, Centre Hospitalier Universitaire Mohamed VI, Université Mohamed I^er^, Oujda, Maroc

**Keywords:** Tumeur brune, hémodialysée chronique, ostéoporose, brown tumor, chronic hemodialysis, osteoporosis

## Abstract

Les tumeurs brunes constituent une complication rare mais sévère de la dialyse, le plus souvent asymptomatique. Nous rapportons le cas d'une patiente dont le mode de révélation de la tumeur brune fut atypique. Observation médicale: Madame F. est une patiente hémodialysée chronique depuis 14 ans suite à une néphropathie indéterminée. Sur le plan phosphocalcique, elle présente une ostéoporose depuis plus de 10 ans, ainsi qu'une hyperparathyroïdie secondaire. A la date du 24 octobre 2013, elle rapporte qu'elle a constaté la présence d'une petite « bosse » pariétale, apparue de façon spontanée la veille, sans notion de traumatisme. A l'examen clinique, nous trouvons une petite tuméfaction de 2cm de diamètre environs, de consistance dure, indolore, mobile par rapport au cuir chevelu. L’échographie a conclu à un lipome. Le lendemain matin, la patiente entre dans un état de coma profond. Le scanner cérébral a révélé la présence d'un hématome extradural associé à un hématome superficiel extra-crânien en regard d'une lyse de l'os pariétal, qui est en fait une tumeur brune. La patiente est transférée en réanimation où elle décède dans la journée.

## Introduction

Les tumeurs brunes constituent une complication rare mais sévère de la dialyse. Elles correspondent à des remaniements osseux associés à des microfractures et des saignements intralacunaires secondaires à une hyperparathyroïdie. Elles peuvent être asymptomatiques et ne se manifester que par une tuméfaction plus ou moins volumineuse et gênante ou induire des symptômes telles que des douleurs, des fractures spontanées ou des tableaux neurologiques à type de paraparésies voire paraplégies. Nous rapportons le cas d'une patiente dont le mode de révélation de la tumeur brune fut pour le moins atypique.

## Patient et observation

Madame E.F. est une patiente de 55 ans, non diabétique et non hypertendue, mise en hémodialyse depuis 14 ans suite à une néphropathie indéterminée, à raison de deux séances par semaine. Ses prises de poids interdialytiques habituelles sont comprises entre 1 kg et 1kg 500 et ses tensions artérielles sont de l'ordre de 100/60 mmHg. Elle est porteuse d'une hépatite virale c inactive depuis 8 ans. Sur le plan phosphocalcique, elle présente une ostéoporose depuis plus de 10 ans, ainsi qu'une hyperparathyroïdie secondaire avec un taux de PTH1-84 de 650 pg/ml traitée par du carbonate de calcium et du calcitriol.

A la date du 24 octobre 2013, elle rapporte qu'elle a constaté la présence d'une petite « bosse » pariétale, apparue de façon spontanée la veille, sans notion de traumatisme ni d’évènement particulier.

A l'examen clinique, nous trouvons une petite tuméfaction de 2cm de diamètre environs, de consistance dure, indolore, mobile par rapport au cuir chevelu qui est resté de couleur normale. Durant la séance de dialyse qui a été menée avec l'anticoagulation habituelle à base d'héparine standard à la dose totale de 25 mg, la tuméfaction n'a pas changé de taille ou de consistance. Après le débranchement, nous avons tout de même décidé de réaliser une échographie de la tuméfaction par un opérateur expérimenté qui a conclu à un lipome, que nous avons respecté.

Le lendemain matin, la patiente a été amenée aux urgences par sa famille dans un état de coma profond avec à l'examen clinque un GCS à 3/15. Le scanner cérébral a révélé la présence d'un hématome extra-dural associé à un hématome superficiel extra-crânien en regard d'une lyse de l'os pariétal. En fait, le « lipome » était un hématome secondaire à une tumeur brune responsable d'une lyse osseuse dont les brèches ont lésé les vaisseaux en contact; l'extravasation de sang a été responsable de l'hématome extradural dont une partie s'est issue en dehors du crâne. La patiente a été mise en condition et transférée en unité de soins intensifs; elle est décédée dans la journée.

## Discussion

Les tumeurs brunes sont des tumeurs bénignes retrouvées dans les hyperparathyroïdies primaires ou secondaires. Elles sont observées chez les patients en insuffisance rénale chronique lorsque l'hyperparathyroïdie secondaire est sévère et persistante [1]. Il en résulte des microfractures osseuses localisées et des hémorragies qui provoquent localement l'afflux de macrophages multinucléés (ostéoclastes) et l'apparition d'une fibrose médullaire réactionnelle (stroma de fibroblastes), formant ainsi une masse tumorale. Les lésions, souvent multifocales, se retrouvent le plus souvent au niveau des mandibules, des côtes, des clavicules, du pelvis, du crâne et du massif facial.

Sur le plan clinique, elles sont souvent asymptomatiques ou ont contraire se révèlent par une sensation de gène, des douleurs voire des fractures osseuses. Elles peuvent également conduire à un tableau clinique bruyant de type neurologique. La nature du mode de révélation de la tumeur brune de notre patiente laisse perplexe et pousse bien à tirer deux enseignements majeurs: tout d'abord, aussi peu fréquentes soient-elles, les tumeurs brunes peuvent avoir des conséquences tellement fatales qu'elles ne doivent pas être méconnues; ensuite, il ne faut jamais négliger ou sous-estimer le moindre des symptômes chez les hémodialysés chroniques, aussi anodin puisse-t'il paraitre.

Radiologiquement, les tumeurs brunes se présentent sous forme d'images lytiques de taille variable, uniques ou multiples, souvent excentrées et soufflant la corticale [2]. La réaction périostée et l'envahissement des tissus mous sont quasi absents, éléments caractéristiques du diagnostic [1]. Le scanner reste l'examen de choix avec un rehaussement possible après injection de produit de contraste, qui ne pose pas de problème chez les hémodialysés chroniques. L'IRM est utile dans les localisations vertébrales. Les lésions osseuses extra-faciales multiples peuvent être recherchées par la scintigraphie osseuse au technétium. Dans le cas de notre patiente, nous avons été surpris par le diagnostic de tumeur brune apporté par le scanner cérébral dont l'indication était la survenue de troubles de conscience d'installation brutale.

Histologiquement, les tumeurs brunes n'ont pas d'aspect spécifique, si bien que seule la coexistence de lésions ostéolytiques multiples et de l'hyperparathyroïdie permet de conclure à leur diagnostic [3]. L'immunohistochimie et les diverses colorations n'ont de plus pas de place dans celui-ci.

## Conclusion

Cette expérience nous a permis de tirer au moins deux enseignements principaux: tout d'abord, qu'il faut accorder une importance primordiale à chaque nouveau symptôme chez le dialysé aussi banal puisse-t-il paraitre car celui-ci peut orienter vers un diagnostic parfois d'issue fatale. Deuxièmement, les tumeurs brunes peuvent se présenter sous forme compliquée d'emblée et la gravité des tableaux qu'elles peuvent entrainer justifie de ne pas les méconnaitre malgré leur faible prévalence.
